# Supplementary material for: Structural Insight into Catalysis by the Flavin-Dependent NADH Oxidase (Pden_5119) of Paracoccus denitrificans
Source: Int J Mol Sci. 2023 Feb 13;24(4):3732. doi: 10.3390/ijms24043732 (PMC9963409; doi:10.3390/ijms24043732)
Supplement: Supplementary file 1 [file ijms-24-03732-s001.zip › ijms-2146169-supplementary.pdf]

Supplementary Materials

Structural Insight into Catalysis by the Flavin-Dependent NADH Oxidase (Pden_5119) of *Paracoccus denitrificans*

Martin Kryl, Vojtěch Sedláček and Igor Kučera *

Department of Biochemistry, Faculty of Science, Masaryk University, Kotlářská 2, 61137 Brno, Czech Republic
* Correspondence: ikucera@chemi.muni.cz; Tel.: +420-54949-5392

Supplementary Figures

Figure S1. Kinetics of diethyl pyrocarbonate hydrolysis in 0.1 M sodium phosphate pH 7.3 at 0 °C.

Figure S2. pH dependence of Pden_5119 inactivation by pyridoxal 5′-phosphate.

Figure S3A. Semi-logarithmic plot of time courses of inactivation of Pden_5119 at different concentrations of 1-acetylimidazole with correction for reagent decomposition.

Figure S3B. Plot of the k_{app} values, derived from the slopes of the lines in (A), versus 1-acetylimidazole concentration.

Figure S4. Kinetics of hydrolysis 1-acetylimidazole (0.2 mM initial) in 0.05 M sodium phosphate pH 7.3 at 30 °C.

Figure S5A. Semi-logarithmic plot of time courses of inactivation of Pden_5119 at different concentrations of phenylglyoxal.

Figure S5B. Plot of the $\mathrm{k_{app}}$ values, derived from the slopes of the lines in (A), versus phenylglyoxal concentration.

Figure S6. Phylogenetic tree showing relationship among proteins structurally similar to Pden_5119.

Figure S7. CD spectra of the wild-type and mutant Pden_5119 proteins.

Figure S8. Dependence of $\mathrm{k_{obs}}$ for the reduction of enzyme-bound FMN on the initial concentration of NADH (left panels) and for the oxidation of the enzyme-bound reduced FMN on the initial concentration of oxygen (right panels).

Figure S9. Semi-logarithmic plot of time courses of inactivation of Pden_5119 wild type and R116A mutant by 40 mM phenylglyoxal.

Supplementary Tables

Table S1. Crystallization parameters of the Pden_5119 protein.

Table S2. Data collection from X-ray diffraction.

Table S3. Structure solution and refinement.

Table S4. Data collection from SAXS.

Table S5. The mutagenic oligonucleotide primers for site-directed mutagenesis of the Pden_5119 protein.

Supplementary Figures

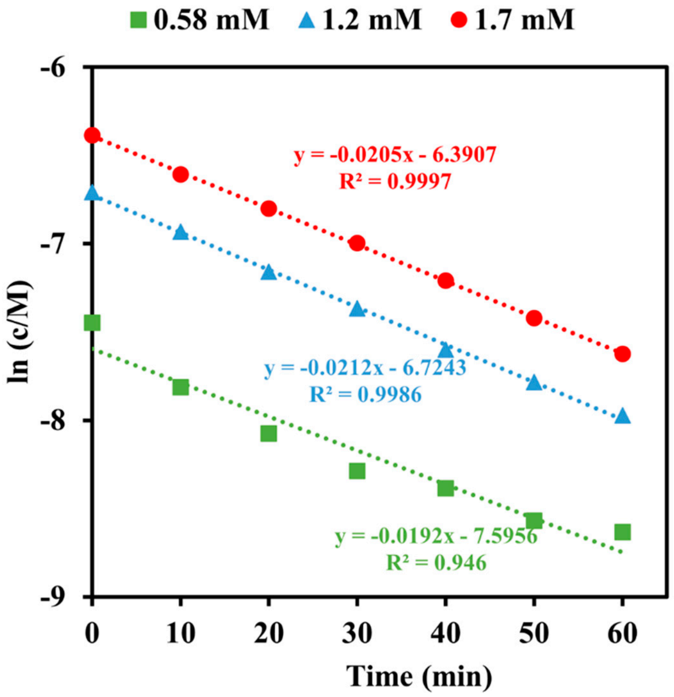

Figure S1. Kinetics of diethyl pyrocarbonate hydrolysis in 0.1 M sodium phosphate pH 7.3 at 0 °C. The experiments were started by adding 50, 100, or 150 µL of 0.12 M ethanolic diethyl pyrocarbonate solution to 10 mL of ice-cold phosphate buffer. At 10-min intervals 200-µL samples were withdrawn, mixed with 800 µL of 12.5 mM imidazole in phosphate buffer and after a half-hour at room temperature the absorbance at 230 nm was measured against 10 mM imidazole solution as a blank. Diethyl pyrocarbonate concentration was calculated using the molar absorption coefficient of 3000 $M^{-1}\cdot cm^{-1}$ and the dilution factor of 5. The linearity of semi-logarithmic plots of the concentration-time courses confirms the first-order kinetics. By averaging the values of slopes of the plots, the final value of the rate constant k´ was obtained to be 0.0203 min^{-1}.

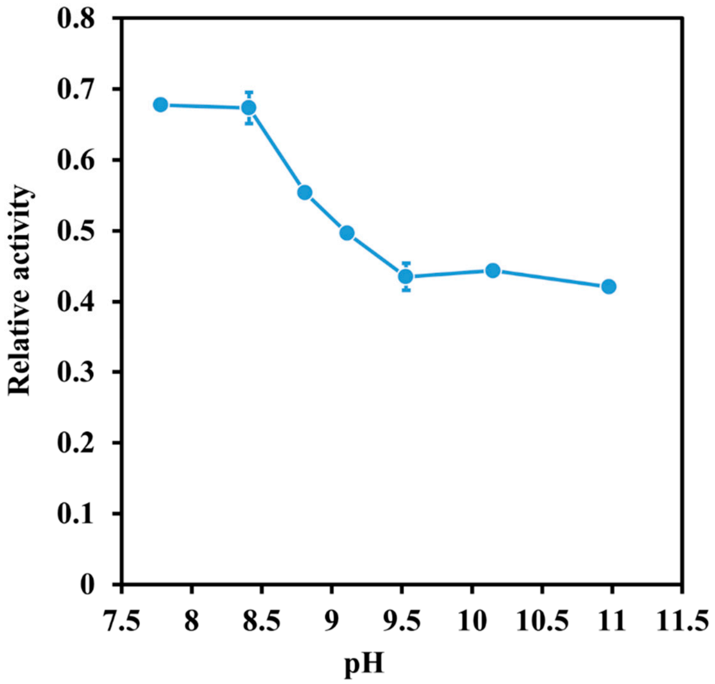

Figure S2. pH dependence of Pden_5119 inactivation by pyridoxal 5′-phosphate. The inactivation reaction was carried out for 30 min at 30 °C in 40 mM Britton-Robinson buffer of an appropriate pH with 25.2 μM enzyme and 9.1 mM pyridoxal 5′-phosphate. The activities (means ± SEM, n = 3) are normalized relative to controls incubated in buffers without inactivator.

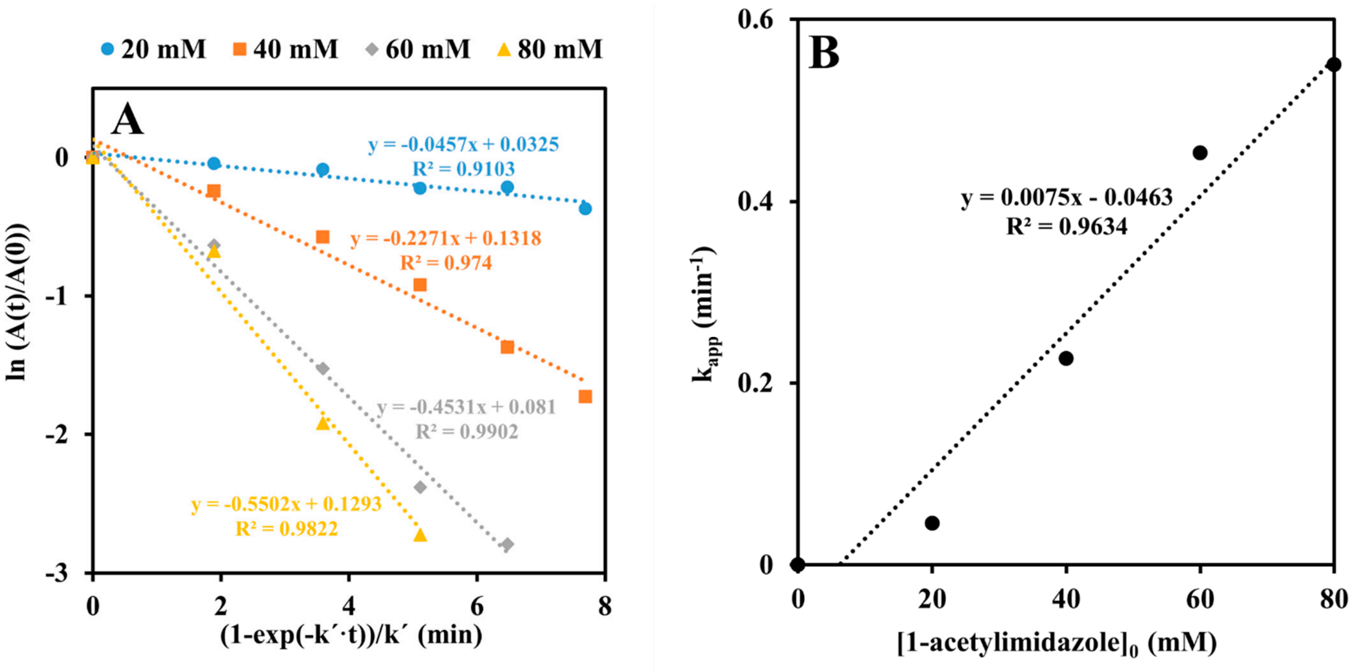

Figure S3. (A) Semi-logarithmic plot of time courses of inactivation of Pden_5119 at different concentrations of 1-acetylimidazole with correction for reagent decomposition. Reactions were performed at 30 °C in 40 μL of 50 mM sodium phosphate buffer, pH 7.3, with 0.11 mM enzyme. Millimolar 1-acetylimidazole concentrations were 20, 40, 60 and 80. 5-μL aliquots were withdrawn at 2-min time intervals and assayed for residual enzymatic activity. No loss of activity was observed during incubation of the enzyme in the absence of 1-acetylimidazole. The first-order rate constant for decomposition of 1-acetylimidazole, k', was determined separately and amounted to 0.055 min^{-1} (see Figure S4). (B) Plot of the k_{app} values, derived from the slopes of the lines in (A), versus 1-acetylimidazole concentration. The rate constant, k, for inactivation is equal to the slope of the regression line which is $7.5 \cdot 10^{-3}$ $mM^{-1} \cdot min^{-1}$ with a standard error of $9 \cdot 10^{-4}$.

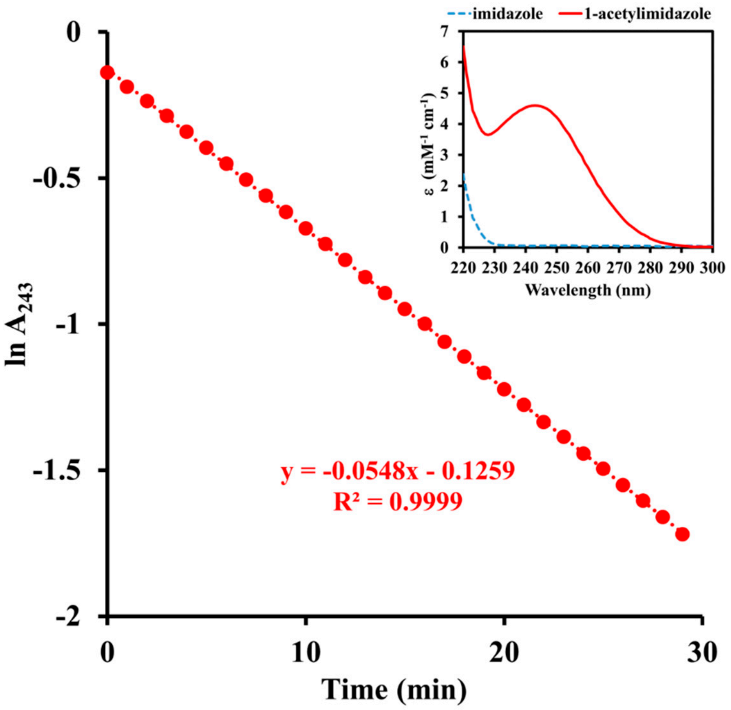

Figure S4. Kinetics of hydrolysis 1-acetylimidazole (0.2 mM initial) in 0.05 M sodium phosphate pH 7.3 at 30 °C. Since 1-acetylimidazole differs from imidazole by exhibiting an absorption band at 243 nm (see the inset), the hydrolysis rate was measured at this wavelength. The first-order rate constant k´ was calculated as the negative slope of the semi-log plot of A_{243} vs time and was found to be 0.055 min^{-1}.

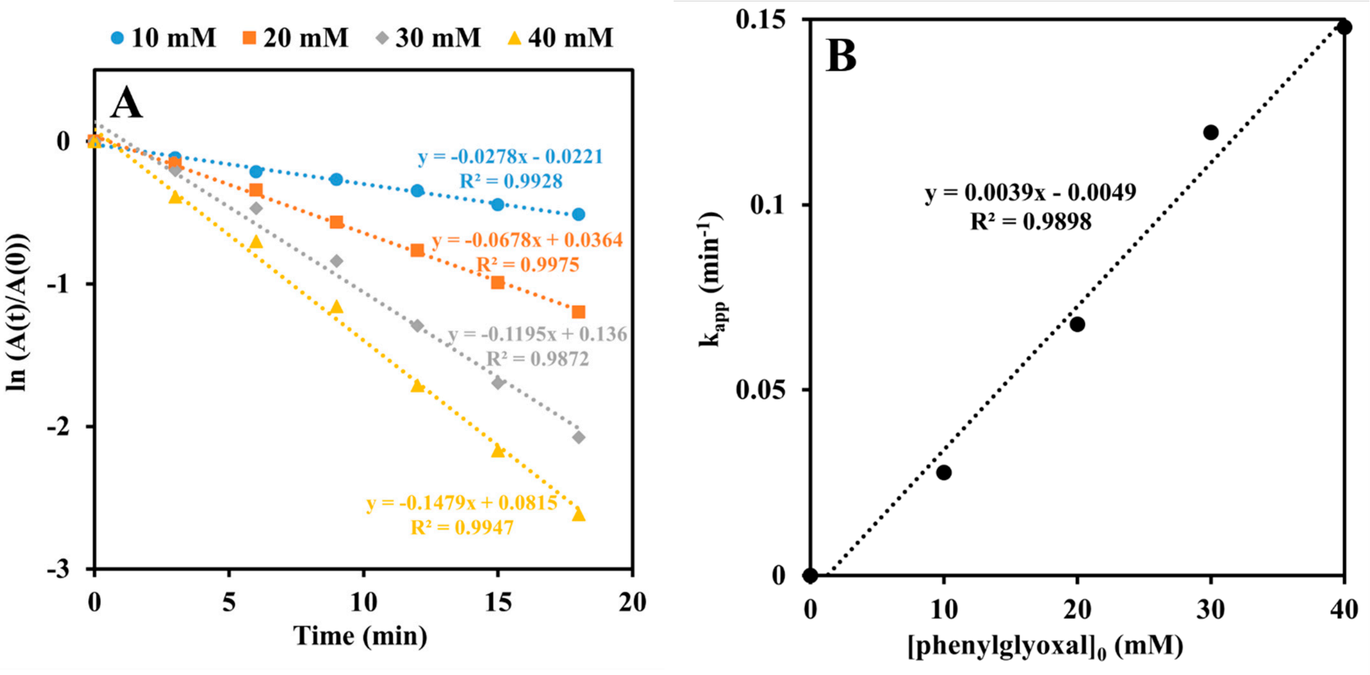

Figure S5. (A) Semi-logarithmic plot of time courses of inactivation of Pden_5119 at different concentrations of phenylglyoxal. Reactions were performed at 30 °C in 40 μL of 0.1 M sodium phosphate buffer, pH 7.3, with 0.16 mM enzyme. Millimolar phenylglyoxal concentrations were 10, 20, 30 and 40. Five microliters aliquots were withdrawn at 3-min time intervals and assayed for residual enzymatic activity. No loss of activity was observed during incubation of the enzyme in the absence of phenylglyoxal. (B) Plot of the k_{app} values, derived from the slopes of the lines in (A), versus phenylglyoxal concentration. The rate constant, k, for inactivation is equal to the slope of the regression line which is $3.9 \cdot 10^{-3}$ $mM^{-1} \cdot min^{-1}$ with a standard error of $2 \cdot 10^{-4}$.

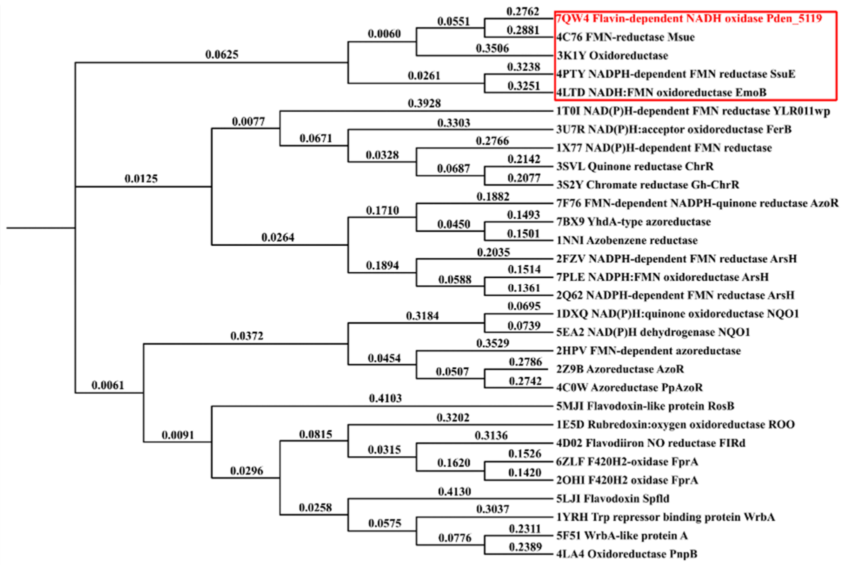

Figure S6. Phylogenetic tree showing relationship among proteins structurally similar to Pden_5119. Alignment of the amino acid sequences was performed and the phylogenetic tree was constructed by Clustal Omega (http://www.ebi.ac.uk/Tools/msa/clustalo/), with the default settings. Distance measures between leaves of the tree are listed above branches. Further consideration was focused on proteins in the red highlighted clade.

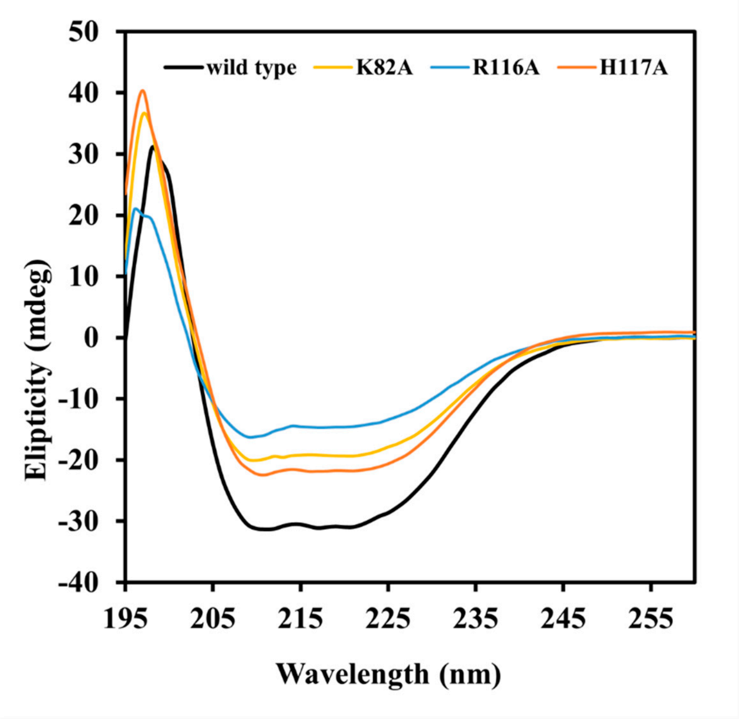

Figure S7. CD spectra of the wild-type and mutant Pden_5119 proteins. The final concentration was 0.3 mg·mL^{-1} (the wild type), 0.2 mg.mL^{-1} (H117A), 0.2 mg.mL^{-1} (K82A), and 0.15 mg.mL^{-1} (R116A) in 0.1 M sodium phosphate buffer (pH 7.0).

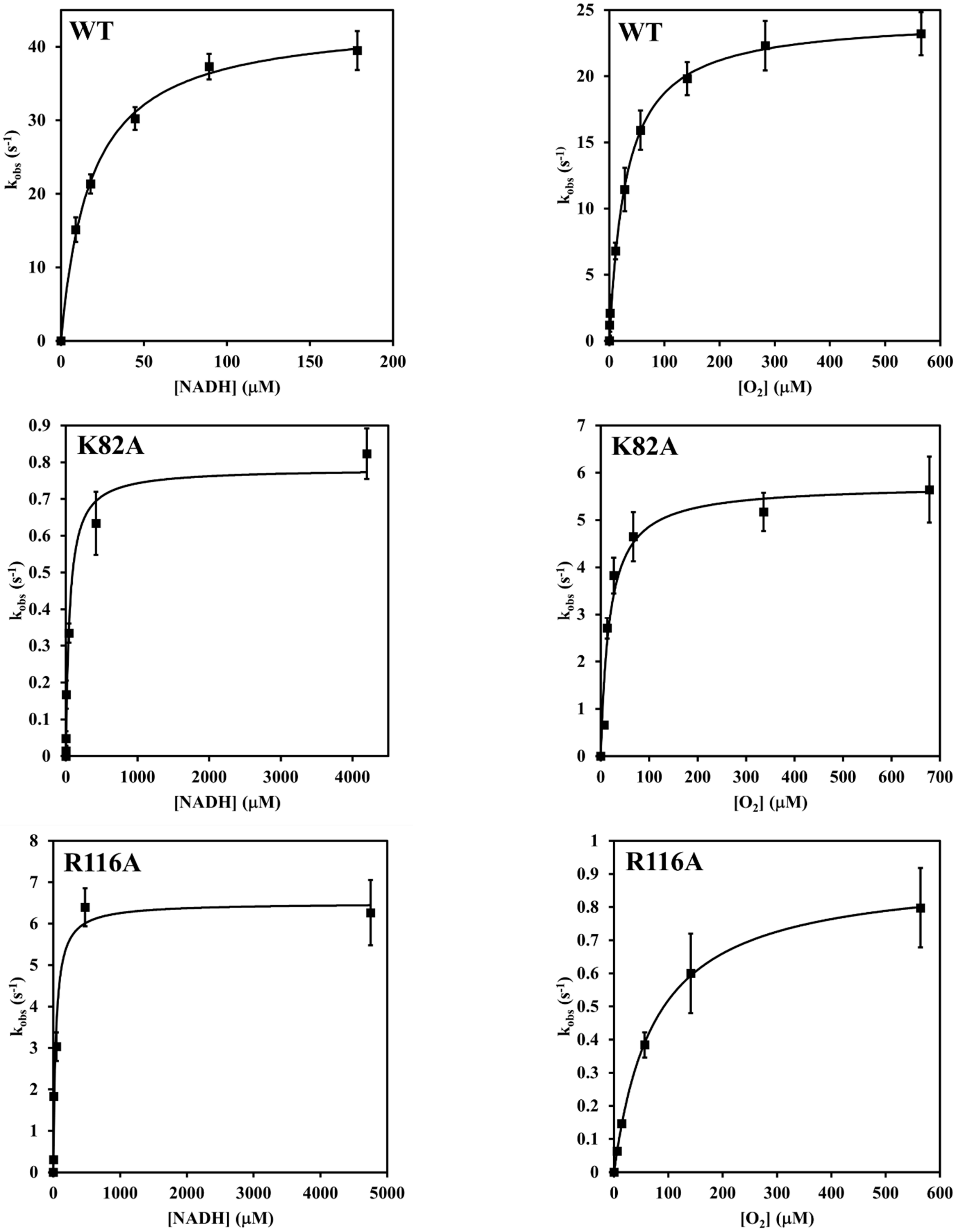
WT
k_{obs} (s^{-1})
[NADH] (μM)
WT
k_{obs} (s^{-1})
[O_2] (μM)
K82A
k_{obs} (s^{-1})
[NADH] (μM)
K82A
k_{obs} (s^{-1})
[O_2] (μM)
R116A
k_{obs} (s^{-1})
[NADH] (μM)
R116A
k_{obs} (s^{-1})
[O_2] (μM)

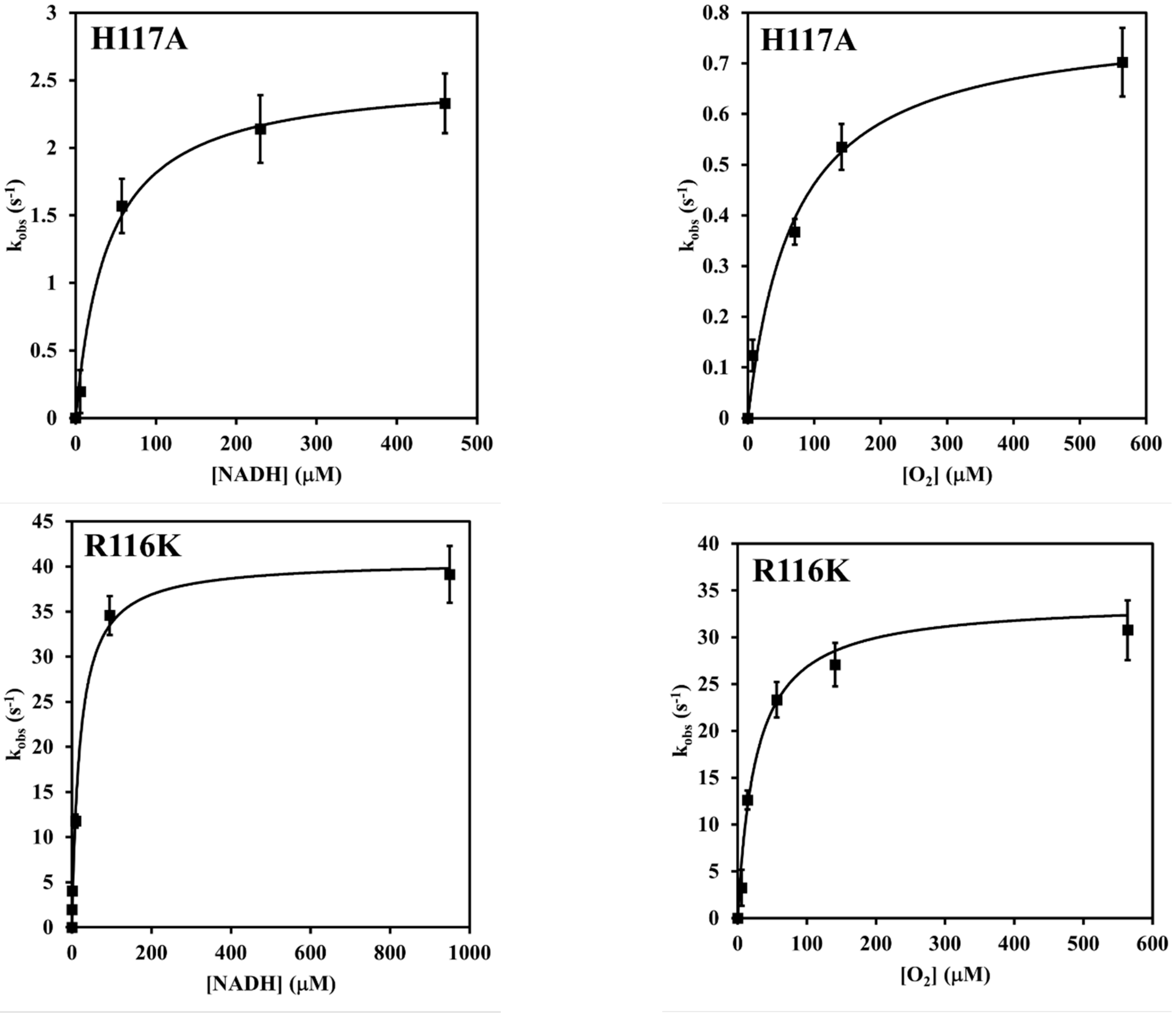

Figure S8. Dependence of k_{obs} for the reduction of enzyme-bound FMN on the initial concentration of NADH (left panels) and for the oxidation of the enzyme-bound reduced FMN on the initial concentration of oxygen (right panels). The final concentration of the wild type protein, K82A, R116A, H117A, and R116K with FMN after mixing in the measuring cell was 50 µM, 43 µM, 57 µM, 54 µM, and 46 µM, respectively. Data are means ± SD, n = 6-9. The regression curves have the equation $k_{obs} = k_{red} \cdot [NADH]/(K_{NADH} + [NADH])$ (left panels) or $k_{obs} = k_{ox} \cdot [O_2]/(K_{O2} + [O_2])$ (right panels).

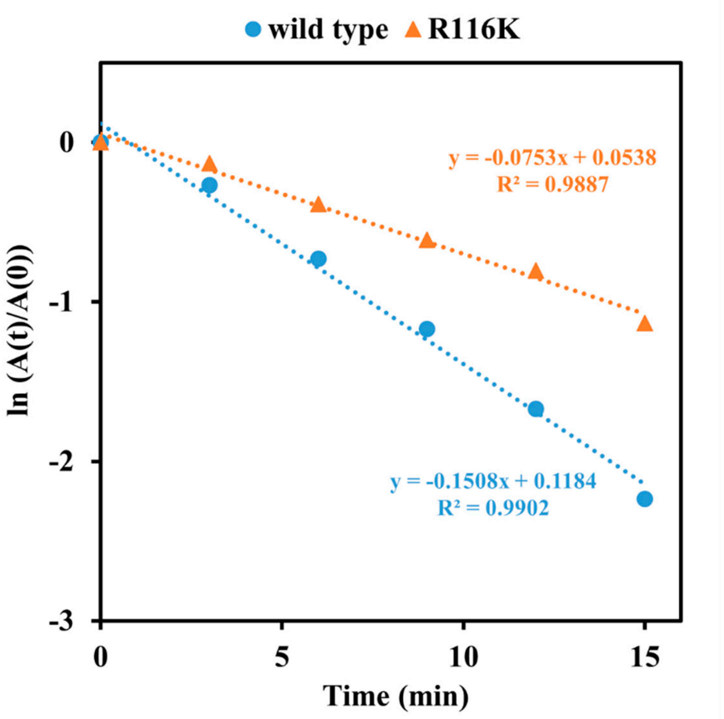

Figure S9. Semi-logarithmic plot of time courses of inactivation of Pden_5119 wild type and R116A mutant by 40 mM phenylglyoxal. Conditions of experiments were as described in the legend to the Figure S5.

Supplementary Tables

Table S1. Crystallization parameters of the Pden_5119 protein.

Method	Vapour diffusion in hanging drop configuration
Plate type	Invoplate SD 2 (Molecular Dimensions)
Temperature (K)	298.15
Protein concentration	22
Buffer composition of protein solution	50 mM trisodium phosphate, 300 mM sodium chloride, 300 mM imidazole, pH 8.0
Composition of reservoir solution	40% (*v*/*v*) polyacrylic acid (MW = 2300) sodium salt in 0.1 M HEPES pH 6.5
Volume and ratio of drop	2000 nL, 1:1
Volume of reservoir	1 mL

Table S2. Data collection from X-ray diffraction.

Diffraction source	SOLEIL BEAMLINE SIGMA
Wavelength (Å)	0.97857
Temperature (K)	100
Detector	PILATUS 3S 6M
Crystal-detector distance (mm)	578.5
Rotation range per image (°)	0.1
Total rotation range (°)	0-360
Exposure time per image (s)	0.1
Space group	19
a, b, c (Å)	134.51, 136.376, 221.314
α, β, γ (°)	90, 90, 90
Mosaicity (°)	0.25
Resolution range (Å)	48.75-3.26
Total No. of reflections	128783
No. of unique reflections	64509
Completeness (%)	90.22
Redundancy	2
$\langle I/\sigma(I) \rangle$	9.7 (2.26)
$R_{r.i.m.}$	0.0603
Overall B factor from Wilson plot (Å^2)	86

Table S3. Structure solution and refinement.

Resolution range (Å)	48.75–3.1
Completeness (%)	90.19 (99.96)
σ cutoff	F > 1.440σ(F)
No. of reflections, working set	64467(4884)
No. of reflections, test set	4885(159)
Final R_{cryst}	0.3201 (0.3292)
Final R_{free}	0.3212(0.3157)

Table S4. Data collection from SAXS.

**Data-collection parameters**	
Instrument	BioSAXS-1000 (Rigaku)
Wavelength [Å]	1.5418
q range [$Å^{-1}$]	0.015 - 0.65
Exposure time [min]	300
Temperature [°C]	4
Concentration [mg·mL^{-1}]	3.0 and 1.5 (merged)
**Structural parameters**	
I(0) [a.u.] from Guinier	0.14
Rg [Å] from Guinier	23.4
I(0) [a.u.] from P(r)	0.14
Rg [Å] from P(r)	23.0
D_{max} [Å]	62.68
Porod volume estimate [$Å^3$]	69800
MW theoretical [kDa] – monomeric unit	20.6
MW porod [kDa]	43.6
MW dammif [kDa]	38.3

Table S5. The mutagenic oligonucleotide primers for site-directed mutagenesis of the Pden_5119 protein. FP - forward primer, RP - reverse primer.

Primers	Sequence
K82A-FP	5′-CGG TAT AGC TGC CCG CGT AGA CGG GGC TGG-3′
K82A-RP	5′-CCA GCC CCG TCT ACG CGG GCA GCT ATA CCG-3′
R116A-FP	5′-ACC AGC GCA TGA GCG TCC CCG CCC CC-3′
R116A-RP	5′-GGG GGC GGG GAC GCT CAT GCG CTG GT-3′
R116K-FP	5′-GAT CAC CAG CGC ATG CTT GTC CCC GCC CCC GGT-3′
R116K-RP	5′-ACC GGG GGC GGG GAC AAG CAT GCG CTG GTG ATC-3′
H117A-FP	5′-CCA GCG CAG CAC GGT CCC CGC CCC C-3′
H117A-RP	5′-GGG GGC GGG GAC CGT GCT GCG CTG G-3′
